# Supplementary material for: Phenotype prediction from single-cell RNA-seq data using attention-based neural networks
Source: Bioinformatics. 2024 Feb 23;40(2):btae067. doi: 10.1093/bioinformatics/btae067 (PMC10902676; doi:10.1093/bioinformatics/btae067)
Supplement: btae067_Supplementary_Data [file btae067_supplementary_data.pdf]

Supplementary Meterials to ScRAT: Early Phenotype Prediction From Single-cell RNA-seq Data using Attention-Based Neural Networks

Yuzhen Mao[1,*] Yen-Yi Lin[2,3,*] Nelson K.Y. Wong[4] Stanislav Volik[3]
Funda Sar[2,3] Colin Collins[2,3,†]
Martin Ester[1,3,†]
[1]School of Computing Science, Simon Fraser University, Burnaby, Canada, V5A 1S6
[2]Department of Urologic Sciences, University of British Columbia, Vancouver, Canada, V5Z 1M9
[3]Vancouver Prostate Centre, Vancouver, Canada, V6H 3Z6
[4]Department of Experimental Therapeutics, BC Cancer, Vancouver, Canada, V5Z 1L3

Contents

1 Summary of COVID scRNA-seq Atlases **2**
1.1 Distribution of Clinical Variables Among Patients 3

2 Experimental Design **5**
2.1 Baselines 5
2.2 Configuration of ScRAT 5

3 Extend ScRAT to Multi-level and Continuous Trait Prediction **5**

4 Ablation Study **5**

5 Evaluations of Phenotype Prediction Accuracy **7**

6 Evaluations of Phenotype Prediction across Different Datasets **7**

7 Distribution of High-Attention Cells in the Haniffa Dataset **9**

8 Comparisons of Performance using PCA before and after data splitting **10**

*These authors contributed equally to this work.
†To whom correspondence should be addressed. Email: colin.collins@ubc.ca, ester@sfu.ca

1 Summary of COVID scRNA-seq Atlases

This section provides the breakdown of samples from each atlas in our experiment. A single patient can be sampled multiple times and contribute to multiple samples. Since we exclude samples with less than 500 cells, we define *effective samples* as those samples with at least 500 cells, and *effective cells* as the collections of all cells from effective samples used in our experiments.

Dataset	Phenotype	#Cells	#Samples	#Samples in Class 1	#Samples in Class 2
Combat	Disease Diagnosis	835,937	121	77	44
Haniffa	Disease Diagnosis	528,438	105	71	34
SC4	Disease Severity	501,943	91	29	62
	Disease Stage	1,289,496	229	138	91

Table 1: **Summary of 3 Datasets.** We exclude any samples with less than 500 cells or unclear clinical phenotype annotations from the original datasets. Note that one patient can be sampled multiple times and contribute to multiple samples. For Combat and Hannifa, the numbers of samples in Class 1 and 2 correspond to the number of COVID and non-COVID samples. Class 1 and 2 in SC4 correspond to mild/moderate vs severe/critical phenotypes among 91 samples of progression for the severity prediction, and convalescence vs progression for the stage prediction. See Supplementary Table 2 - 4 for detailed information.

Labels	COVID status	Annotations	Number of Samples	Number of Cells	Number of Samples < 500 cells	Number of effective samples	Number of effective cells
HV	No	Healthy	10	92,205	0	10	92,205
FLU	No	Influenza Acute	12	19,233	1	11	19,058
Sepsis	No	Sepsis acute(IP)	23	164,128	0	23	164,128
COVID_MILD	Yes	Mild	17	114,418	0	17	114,418
COVID_SRV	Yes	Severe	30	247,799	0	30	247,799
COVID_CRT	Yes	Critial	17	93,982	1	16	93,969
COVID_LDN	Yes	Low-dose Naltrexone	2	15,485	0	2	15,485
COVID_HCW_MILD	Yes	Community COVID-19	13	88,898	1	12	88,875

Table 2: Overview of Combat scRNA-seq dataset.

Labels	COVID status	Annotations	Number of Samples	Number of Cells	Number of Samples < 500 cells	Number of effective samples	Number of effective cells
Healthy	No	Healthy	24	97,039	0	24	97,039
LPS	No	LPS	12	7,884	6	6	6,403
Non-covid	No	Non-covid	5	15,157	0	5	15,157
Asymptomatic	Yes	Asymptomatic	12	33,601	1	11	33,227
Critical	Yes	Critical	16	63,854	0	16	63,854
Mild	Yes	Mild	19	93,835	0	19	93,835
Moderate	Yes	Moderate	29	179,012	1	28	178,688
Severe	Yes	Severe	7	40,235	0	7	40,235

Table 3: Overview of Hannifa scRNA-seq dataset.

Labels	Number of Samples	Number of Cells	Number of Samples < 500 cells	Number of effective samples	Number of effective cells
Disease Stage					
convalescence	140	787,987	2	138	787,553
progression	116	509,715	25	91	501,943
Disease Severity					
mild/moderate	33	164,286	4	29	162,741
severe/critical	83	345,429	21	62	339,202

Table 4: Overview of SC4 scRNA-seq dataset.

1.1 Distribution of Clinical Variables Among Patients

The distribution of age among COVID and Non-COVID patients can be found in Table 5, and the distribution of sex among 2 phenotypes in Table 6. For each public dataset we randomly split patients into training and testing in each iteration, therefore the sampling results should reflect the overall trends in the original datasets. We believe that this is the best available strategy to minimize the potential impacts driven by other confounding factors.

	Hannifa		SC4	
Age	Percentage among COVID patients	Percentage among Non-COVID patients	Percentage among COVID patients	Percentage among Non-COVID patients
≤ 30	3.9%	24.4%	10.5%	11.9%
$30 \sim 39$	7.8%	26.8%	19.3%	22.4%
$40 \sim 49$	18.6%	7.3%	18.1%	28.4%
$50 \sim 59$	26.5%	21.9%	19.9%	34.3%
$60 \sim 69$	17.6%	12.2%	17.0%	1.5%
$70 \sim 79$	16.6%	7.4%	11.1%	1.5%
≥ 80	9%	0%	4.1%	0%

Table 5: Distribution of Age for COVID and Non-COVID patients in the Haniffa and SC4 Datasets.

	Hannifa		SC4	
Sex	Percentage among COVID patients	Percentage among Non-COVID patients	Percentage among COVID patients	Percentage among Non-COVID patients
Male	51.6%	51.4%	61.4%	80.0%
Female	48.4%	48.6%	38.6%	20.0%

Table 6: Distribution of Sex for COVID and Non-COVID patients in the Haniffa and SC4 Datasets.

2 Experimental Design

2.1 Baselines

For pseudo-bulk baseline methods, we first average the gene expression across all cells in one sample to simulate a pseudo bulk assay as the input to the prediction model. We choose naive linear layer and feed-forward layer as two such prediction models which are denoted as **Linear** and **Feedforward (bulk)** respectively in this paper.

For single-cell methods, single-cell resolution information can be used by two different strategies, either processing each cell separately, or processing all cells in one sample interactively. Simple models such as linear layer and feed-forward layer can be only used for the first strategy since their weights are position-specific, the prediction results change according to the order of cells, which makes it difficult to process multiple cells as a whole. In the experiment, we use feed-forward layer for this strategy and denote this baseline as **Feedforward (single)**. For the second strategy of interactively analyzing all cells, the encoding of a given cell will be affected by others in the same sample and can potentially capture correlations between cells. Vanilla attention layer (Vaswani *et al.*, 2017) and CloudPred (He *et al.*, 2021) are selected as the methods for this strategy, which are denoted as **Attention** and **CloudPred**. We set 10 as the number of clusters in Cloudpred since it achieves the highest AUC among most of the experiments compared with other numbers of clusters we try (5 and 20). Notably, baseline Attention is equivalent to using the ScRAT without sample mixup module.

2.2 Configuration of ScRAT

We have tried several different NC, NS, K combinations, and found that after reaching some thresholds, the difference in the accuracy is marginal and this scenario is consistent across all four datasets. For the simplicity of presentation without hurting the performance, we pick the same group of hyper-parameters that works well for all four datasets as follows.

- We tried $NC = \{200, 400, 500, 600, 800, 1000\}$, and validation results are stable after NC=500.
- We tried $NS = \{10, 20, 40, 50, 60, 80, 100\}$, and validation results are stable after NS=20 for training, NS=50 for validation.
- We tried $K = \{2, 4, 6, 8, 12, 16\}$, and validation results are stable after K=8.

3 Extend ScRAT to Multi-level and Continuous Trait Prediction

ScRAT can be easily extended to handle multi-label prediction or continuous label prediction (regression task). Specifically, for multi-label prediction, one can simply change the output dimension of the output layer to the number of the multi-labels, and use the cross-entropy loss function to calculate the loss. For continuous label prediction, one can change the output dimension of the output layer to one, and use the Mean Squared Error (MSE) loss function to calculate the loss.

4 Ablation Study

Impact of Mixup Strategies. To investigate the impacts of applying mixup only to cells from the same population, we use the Haniffa dataset to compare the performance between ScRAT (i.e., mixup of cells from the same population) and an alternative method that applies mixup to random pairs of cells regardless of their cell populations. The results are shown in Fig. 2(a). Following the predefined clusterings of 9/18/12 cell populations in Combat/Haniffa/SC4, sample mixup can improve the phenotype prediction performance and increases the AUC by up to 1.4% compared to the alternative method, which indicates the efficacy of applying mixup to cells from the same population.

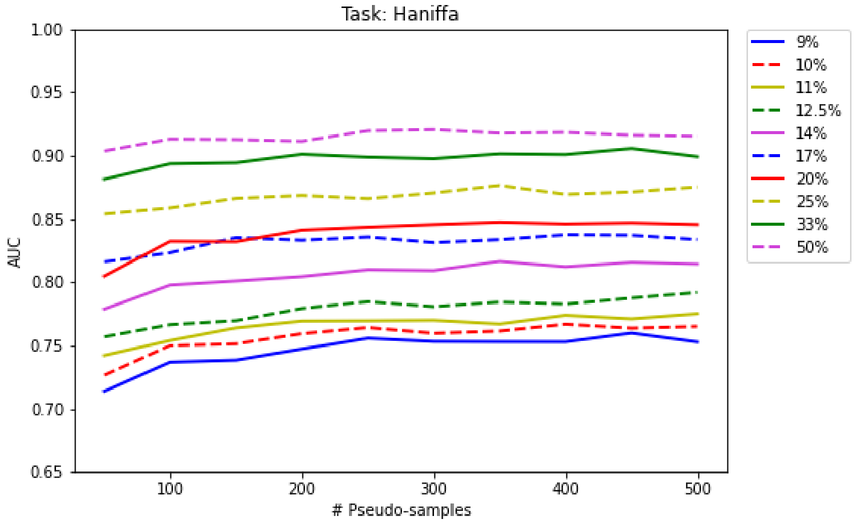

Figure 1: **Impact of the number of the pseudo-samples on ScRAT AUC.** Our experimental results indicate that ScRAT's performance (AUC) is not significantly impacted by the number of pseudo-samples generated by Sample Mixup once it exceeds 250.

What's more, given the number of cells in these datasets, these clusterings are considered low-resolution and can be achieved automatically without human intervention. This indicates that the sample mixup of ScRAT has a minimal dependency of accurate cell type annotations, and can avoid the common challenges of finding the best resolution in scRNA-seq analysis. Moreover, our experiment shows that even without any predefined population information, the random mixup can still be applied to overcome the bottleneck of a limited number of training samples without dramatically hurting the performance.

Impact of Attention Weights. Despite the wide discussions (Serrano and Smith, 2019; Jain and Wallace, 2019; Wiegreffe and Pinter, 2019), the usefulness of the attention mechanism in interpretability is still controversial. Inspired by a recent work about top k attention weights (Gupta *et al.*, 2021), we provide a new perspective for the attention mechanism interpretation by building a bridge between attention weights and the model performance. We design the following two experiments to empirically achieve this goal: given a sample with N cells, we modify the $N \times N$ self-attention matrix described in Eq.3 in two different ways:

Top k: For each row of the matrix, we only keep entries with top k largest attention weights, denoted as $(a_1, \ldots, a_k)$, and set attention values of all remaining entries uniformly as $\frac{1-\sum_{j=1}^{k} a_j}{N-k}$.

Random k: For each row of the matrix, we only keep attention weights for k randomly selected entries, denoted as $(a'_1, \ldots, a'_k)$, and set the attention values of remaining entries uniformly as $\frac{1-\sum_{j=1}^{k} a'_j}{N-k}$.

The results of the above two experiments on Haniffa dataset are presented in Fig. 2(b), where we set k to 5. The AUC of the top k attention method is almost the same as the vanilla attention, which indicates that the top k attention weights are sufficient for the prediction task. On the other hand, the performance of the random k method drops significantly, suggesting the necessity of keeping high attention weights. In this way, we empirically prove the connections between the attention weights and the model performance, and provide an attention mechanism interpretation for ScRAT.

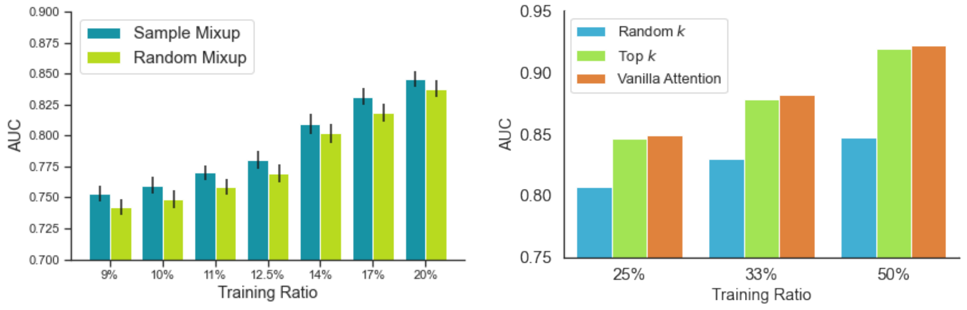

Figure 2: **Ablation Study. Left(a):** *Sample Mixup* corresponds to the current procedure of ScRAT that only mixes cells from the same population. *Random Mixup* means that we apply mixup to any pairs of cells. The result indicates the validity of current sample mixup method. **Right(b):** We compare the performance of various attention matrix re-construction strategies to show that attention weights are related to the model performance. For each row of the attention matrix, *Top* k keeps the top k largest attention weights, and *Random* k keeps k randomly selected attention weights, before normalizing the matrix for the remaining fields. Here, k is set to 5. The results of ScRAT using the original attention matrix described in Eq. 3 is denoted by *Vanilla Attention*. The result shows that top k method achieves AUC comparable to using all attention weights, and they are both better than random k attention. This provides strong evidence of the connection between high attention weights and model performance.

5 Evaluations of Phenotype Prediction Accuracy

We provide the precision and recall for different methods on 4 tasks as the complementary information to AUC values described in the main text. Some alternative methods provide better measurements than ScRAT in some specific tasks with some trade-off. For example, Linear provides higher precision in Combat but low recall, and Feedforward (single) provides better recall in most tasks but low precision. These results support that ScRAT provides the best accuracy of phenotype prediction by compromising between precision and recall.

6 Evaluations of Phenotype Prediction across Different Datasets

In addition to evaluating the model performance within a single dataset, it is more meaningful to conduct experiments across different datasets, for example, transferring the knowledge learned from one dataset to another. However, due to the absence of a standardized rule, different datasets usually contain different input feature spaces, making it infeasible to directly train a model that works for all the datasets. Instead, fine-tuning the model pre-trained on one dataset (source dataset) before applying it to another dataset (target dataset) is necessary.

There are two different fine-tuning strategies. One is fine-tuning the entire model, another is only fine-tuning a subset of layers while fixing the parameters of other layers (Kumar *et al.*, 2022; Lee *et al.*, 2022). The latter one is preferred since training or fine-tuning the entire large model, such as a Transformer, can be very time consuming and may also result in catastrophic forgetting (Serra *et al.*, 2018). Therefore, in the following experiments, instead of updating all the parameters, we choose to only update the first layer (the linear layer) and fix all the layers after it.

To illustrate, we use the Combat dataset as the source dataset for pre-training and the Haniffa dataset as the target dataset for fine-tuning. Our goal is to accurately predict the phenotype of samples in the target dataset. Specifically, we first use the full labeled data from the source dataset to pre-train the model for 100 epochs on the Combat dataset. Afterwards, we freeze all the parameters except the first layer, and

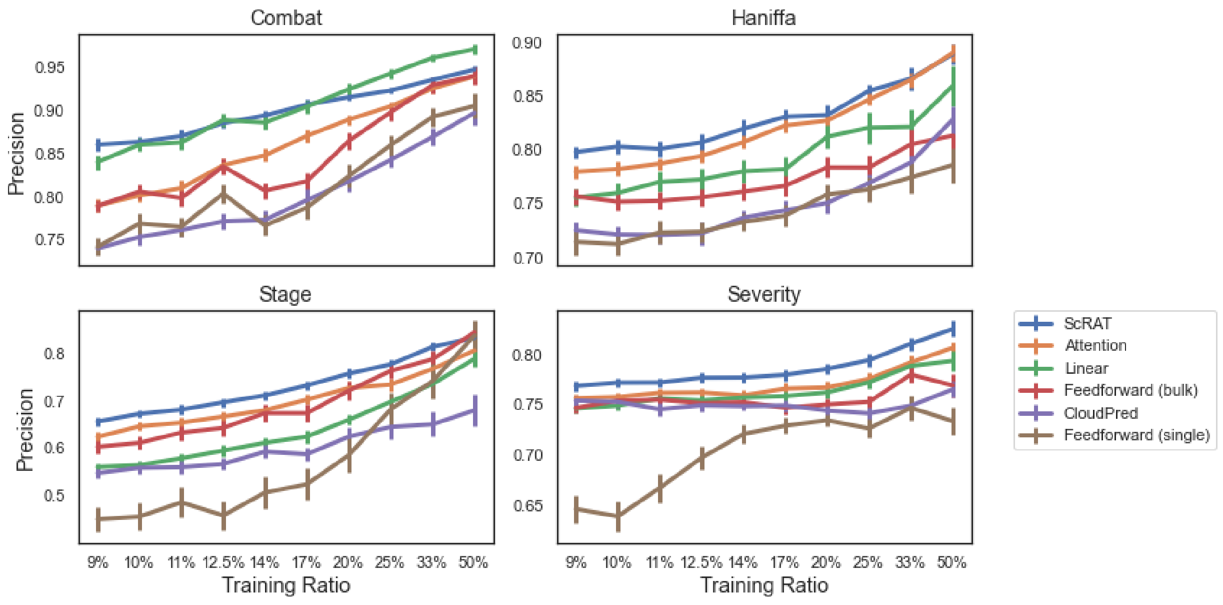

Figure 3: **Comparison of different methods on four different tasks based on precision.** For each task, we report the prediction accuracy of all methods using precision for 10 different training ratios. ScRAT outperforms other methods in all settings with one exception on the Combat dataset.

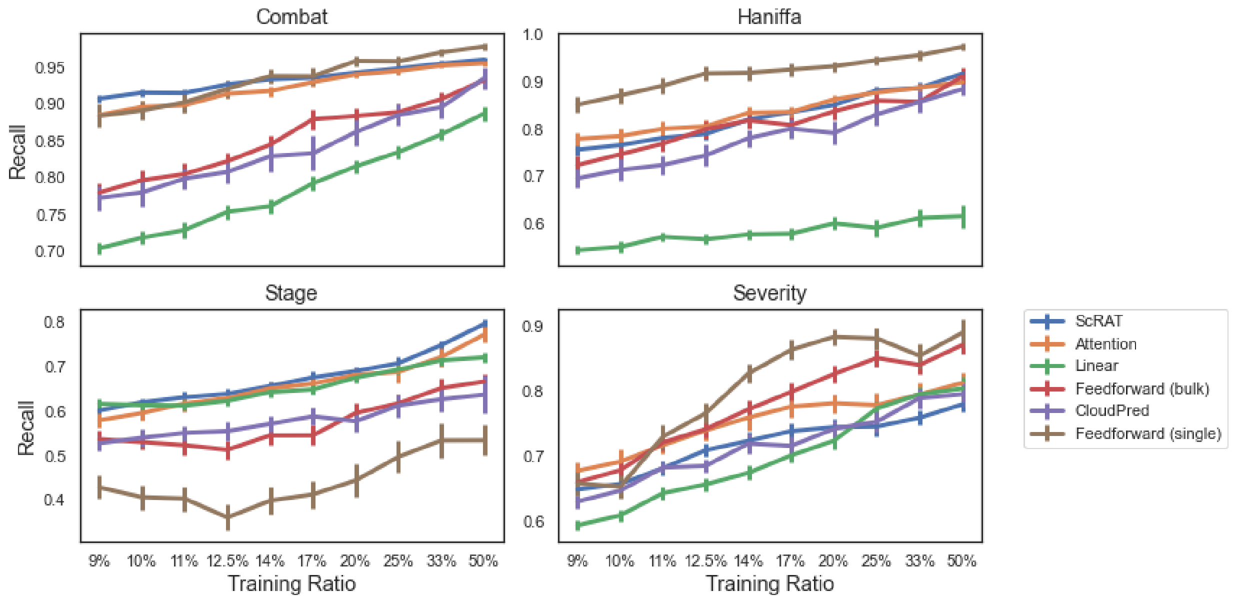

Figure 4: **Comparison of different methods on four different tasks based on recall.** For each task, we report the prediction accuracy of all methods using recall for 10 different training ratios. Feedforward (single) provides the best recall rates but low precision. This suggests the necessity of simultaneously considering information from all cells to better predict the phenotype of a sample.

fine-tune the model on the target dataset for another 100 epochs with early-stopping. We change the number of samples in the target dataset ranging from 9% to 50% of the total number of samples, which aims to simulate the real situation where limited labeled samples are available. We compare the model performance of the transfer-learning experiment with the one of the original non-transfer-learning experiment, and present the corresponding results in Fig. 5.

Based on the results, the transfer-learning experiment (denoted as "Transfer") has a very close performance compared with the non-transfer-learning experiment (denoted as "Attention") across all nine training ratios, and sometimes can even achieve better performance. This result indicates that the knowledge learned from the source dataset using ScRAT can be successfully transferred to the target dataset by fine-tuning only the first layer of the model.

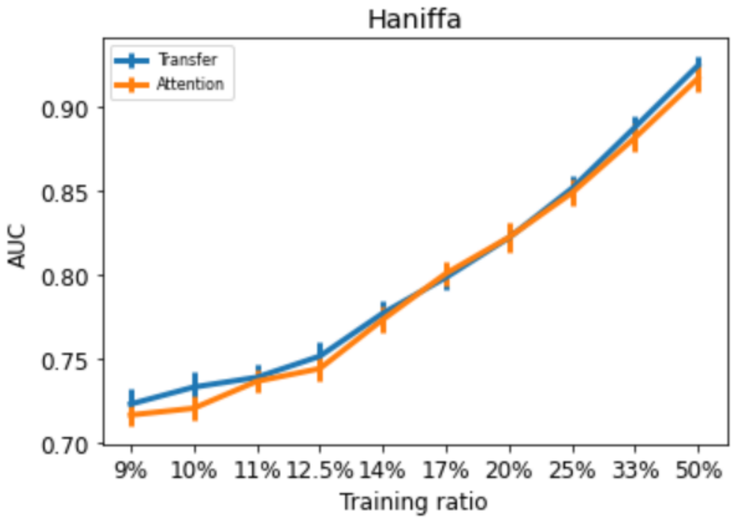

Figure 5: **Transfer knowledge from Combat to Haniffa.** We report the AUC score of ScRAT for 10 different training ratios.

7 Distribution of High-Attention Cells in the Haniffa Dataset

In addition to the *High-attention Occurrence Value* (HOV) discussed in the main text, we also provide the number of high-attention cells for each cell type of the Haniffa dataset in Table 7. Some critical cell types, such as DC3 or CD83_CD14_mono, getting higher rank based on the R-score compared to the one using the ratio of high-attention cells (see Table 1 of the main text). This indicates that high-attention cells in these cell types are getting high-attention values more often than average. Comprehensive analysis of high-attention cells from these cell types will be the most important goal in the next stage of development of ScRAT.

8 Comparisons of Performance using PCA before and after data splitting

In Figure 8 we provide the results of ScRAT on the Hannifa dataset using (1) PCA calculated on the training set and the testing set separately, and (2) PCA calculated on the whole dataset (i.e., results in the current manuscript). The differences show that information leakage is minimal in our current settings.

**High-Attention Cells in the Haniffa Dataset**							
	Number of Cells		Number of High-Attention Cells		Ratio of High-Attention Cells in a Cell Type		
Cell Type	NonCOVID	COVID	NonCOVID	COVID	NonCOVID	COVID	Overall
Platelets	2,899	9,645	903	4348	0.3115	0.4508	0.4186
RBC	307	1,652	156	630	0.5081	0.3814	0.4012
pDC	678	3,384	334	1,083	0.4926	0.3200	0.3488
C1_CD16_mono	198	3,825	61	1,068	0.3081	0.2792	0.2806
Plasmablast	97	3,477	22	899	0.2268	0.2586	0.2577
CD4.Prolif	44	494	14	111	0.3182	0.2247	0.2323
HSC_erythroid	101	608	37	123	0.3663	0.2023	0.2257
Plasma_cell_IgG	171	2,722	35	574	0.2047	0.2109	0.2105
HSC_prolif	7	158	3	30	0.4286	0.1899	0.2000
HSC_MK	0	46	0	9	0.0000	0.1957	0.1957
HSC_CD38pos	87	1,458	24	266	0.2759	0.1824	0.1877
HSC_CD38neg	17	461	3	80	0.1765	0.1735	0.1736
Plasma_cell_IgA	182	1,947	61	291	0.3352	0.1495	0.1653
B_immature	959	3,249	208	470	0.2169	0.1447	0.1611
B_exhausted	552	1,855	75	311	0.1359	0.1677	0.1604
B_non-switched_memory	745	1,940	119	295	0.1597	0.1521	0.1542
NK_prolif	273	3,897	57	539	0.2088	0.1383	0.1429
CD8.Prolif	95	1,084	18	143	0.1895	0.1319	0.1366
DC3	975	2,134	130	270	0.1333	0.1265	0.1287
ASDC	16	79	3	9	0.1875	0.1139	0.1263
B_switched_memory	1,450	4,666	200	544	0.1379	0.1166	0.1216
NK_56hi	2,429	6,431	248	699	0.1021	0.1087	0.1069
CD83_CD14_mono	6,772	42,403	1,053	4,034	0.1555	0.0951	0.1034
DC2	1,052	1,981	136	176	0.1293	0.0888	0.1029
HSC_myeloid	4	46	1	4	0.2500	0.0870	0.1000
Plasma_cell_IgM	88	826	11	79	0.1250	0.0956	0.0985
ILC1_3	213	446	17	42	0.0798	0.0942	0.0895
ILC2	36	48	3	4	0.0833	0.0833	0.0833
Treg	78	223	6	19	0.0769	0.0852	0.0831
B_naive	7,181	29,420	824	2165	0.1147	0.0736	0.0817
NKT	987	2,573	93	176	0.0942	0.0684	0.0756
Mono_prolif	4	593	1	39	0.2500	0.0658	0.0670
gdT	5,134	9,261	314	567	0.0612	0.0612	0.0612
MAIT	3,849	6,126	200	385	0.0520	0.0628	0.0586
CD8.EM	7,001	9,376	328	603	0.0469	0.0643	0.0568
CD4.EM	195	1,329	15	68	0.0769	0.0512	0.0545
NK_16hi	17,104	56,053	1,001	2,748	0.0585	0.0490	0.0512
CD8.TE	8,949	29,672	465	1,508	0.0520	0.0508	0.0511
CD14_mono	3,774	49,231	278	1,785	0.0737	0.0363	0.0389
CD16_mono	3,007	11,664	238	328	0.0791	0.0281	0.0386
DC_prolif	6	102	1	3	0.1667	0.0294	0.0370
CD4.Th1	104	291	3	9	0.0288	0.0309	0.0304
CD8.Naive	8,744	18,951	268	505	0.0306	0.0266	0.0279
CD4.Tfh	826	7,482	51	164	0.0617	0.0219	0.0259
CD4.IL22	7,261	10,324	130	325	0.0179	0.0315	0.0259
CD4.Th2	18	28	0	1	0.0000	0.0357	0.0217
CD4.CM	7,745	26,952	159	494	0.0205	0.0183	0.0188
CD4.Naive	16,080	39,678	334	692	0.0208	0.0174	0.0184
DC1	104	240	0	2	0.0000	0.0083	0.0058
CD4.Th17	1	6	0	0	0.0000	0.0000	0.0000

Table 7: **Number of high-attention cells for each cell type in the Haniffa dataset**. Compared to the HOVs that weight each cell using the high-attention *Occurrence*, here we only count the number of high-attention cells for each cell type. In other words, we force the HOVs = 1 for all high-attention cells to understand their distribution. Note that the *NonCOVID*, *COVID*, and *Overall* values in the "Ratio of High-Attention Cells in a Cell Type" section correspond to the ratio of high-attention cells in a cell type for NonCOVID patients, COVID patients, and all patients respectively. The differences between the rankings based on these ratios and the R-score (see Table 1 in the main text) suggest that some specific cell types include cells getting high-attention values more often than average. More analysis of these cells has the potential to improve our understanding of attention mechanisms in the domain of single-cell biology.

**Haniffa Dataset**										
	Training Ratios									
	9%	10%	11%	12.5%	14%	17%	20%	25%	33%	50%
Number of Training Samples	9	11	12	13	15	18	21	26	35	53
AUC										
ScRAT (PCA-b)	0.7532	0.7596	0.7699	0.7806	0.8092	0.8314	0.8456	0.8706	0.8937	0.9207
ScRAT (PCA-a)	0.7607	0.7610	0.7678	0.7894	0.8129	0.8333	0.8418	0.8695	0.9065	0.9219
Precision										
ScRAT (PCA-b)	0.7980	0.8031	0.8010	0.8070	0.8197	0.8309	0.8324	0.8548	0.8662	0.8888
ScRAT (PCA-a)	0.8070	0.8033	0.8003	0.8088	0.8179	0.8267	0.8371	0.8522	0.8658	0.8841
Recall										
ScRAT (PCA-b)	0.7566	0.7662	0.7816	0.7899	0.8203	0.8363	0.8512	0.8822	0.8871	0.9178
ScRAT (PCA-a)	0.7939	0.8078	0.8180	0.8232	0.8391	0.8479	0.8683	0.8813	0.9033	0.9235

Table 8: **The performance of ScRAT on the Hannifa Datasets using different PCA values.** (1) **ScRAT (PCA-a)**: results using PCA calculated on the training and testing datasets separately. (2) **ScRAT (PCA-b)**: results using PCA applied to all cells without splitting the dataset as shown in the current manuscript. The marginal differences suggest that information leakage is minimal.

References

Gupta, A. *et al.* (2021). Memory-efficient transformers via top-k attention. *arXiv preprint arXiv:2106.06899*.

He, B. *et al.* (2021). Cloudpred: Predicting patient phenotypes from single-cell rna-seq. In *PACIFIC SYMPOSIUM ON BIOCOMPUTING 2022*, pages 337–348. World Scientific.

Jain, S. and Wallace, B. C. (2019). Attention is not explanation. *arXiv preprint arXiv:1902.10186*.

Kumar, A. *et al.* (2022). Fine-tuning can distort pretrained features and underperform out-of-distribution. *arXiv preprint arXiv:2202.10054*.

Lee, Y. *et al.* (2022). Surgical fine-tuning improves adaptation to distribution shifts. *arXiv preprint arXiv:2210.11466*.

Serra, J. *et al.* (2018). Overcoming catastrophic forgetting with hard attention to the task. In *International Conference on Machine Learning*, pages 4548–4557. PMLR.

Serrano, S. and Smith, N. A. (2019). Is attention interpretable? *arXiv preprint arXiv:1906.03731*.

Vaswani, A. *et al.* (2017). Attention is all you need. In *Advances in neural information processing systems*, pages 5998–6008.

Wiegreffe, S. and Pinter, Y. (2019). Attention is not not explanation. *arXiv preprint arXiv:1908.04626*.
